# MR Imaging of a Methotrexate-associated Diffuse Large B-cell Lymphoma in the Liver that Regressed without Treatment

**DOI:** 10.2463/mrms.ci.2015-0061

**Published:** 2015-12-28

**Authors:** Shota MORIKAWA, Ayako MORITA, Ritsuko FUJIMITSU, Yoshifumi KUROKI, Hiroshi URAKAWA, Yoshinobu SHINAGAWA, Daisuke MORIHARA, Mikiko AOKI, Kengo YOSHIMITSU

**Affiliations:** 1 Department of Radiology, Faculty of Medicine, Fukuoka University, 7-45-1 Nanakuma, Jonan-ku, Fukuoka 814-0180, Japan; 2 Department of Gastroenterology, Fukuoka University; 3 Department of Pathology, Fukuoka University

**Keywords:** liver, diffuse large B-cell lymphoma, rheumatoid arthritis, methotrexate

## Clinical Imaging

A 59-year-old febrile woman who had been treated with methotrexate (MTX) for rheumatoid arthritis (RA) presented with a small liver nodule measuring 2 cm in diameter, which showed ring-like enhancement on contrast-enhanced abdominal computed tomography (CT). Laboratory data were within normal limits except for C-reactive protein (7.59 mg/dl, the reference range is no more than 0.20 mg/dl). Serological tests for hepatitis B virus and hepatitis C virus were negative. Soluble IL-2 receptor (561 U/ml, the reference range is 122–496 U/ml) was mildly elevated.

**Fig. 1.**
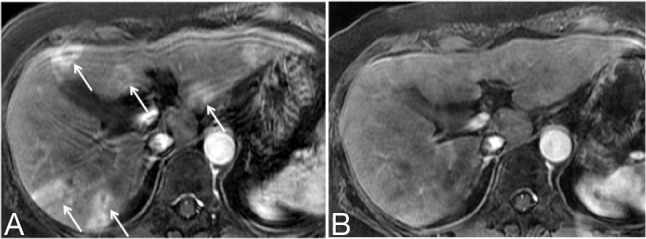
**(A)** Contrast-enhanced T_1_-weighted image (three- dimensional T_1_-weighted gradient-echo image with fat suppression, TR/TE/FA = 4.6 ms/2.2 ms/12°) obtained at arterial phase image using bolus tracking technique after Gd-EOB-DTPA injection obtained before treatment. Multiple liver nodules were detected in the bilateral lobes, which showed ring-like enhancement, especially fused in S6 (arrows). (**B**) Contrast-enhanced T_1_-weighted image obtained at arterial phase image obtained 6 months after discontinuation of MTX. The liver nodules regressed and the ring-like enhancement was unclear.

Follow-up magnetic resonance imaging (MRI) obtained 2 months later revealed multiple larger liver nodules, showing slight T_1_ and T_2_ prolongation and restricted diffusion. On the dynamic phase of Gd-EOB-DTPA (Primovist, Bayer HealthCare, Osaka, Japan) enhancement, the lesions showed persistent ring-like enhancement from the arterial phase to the late phase. On the hepatobiliary phase image, the lesions showed diminished uptake of Gd-EOB-DTPA. 18-fluorodeoxyglucose (FDG) positron emission tomography (PET)/CT showed multiple liver nodules with homogeneous FDG uptake, and no other foci of abnormal uptake. Percutaneous biopsy was attempted for the S6 lesion. Immunohistochemical analysis showed positive immunoreactivity for CD20, but negative for CD3 and CD5, the pathological diagnosis of diffuse large B-cell lymphoma (DLBCL) was obtained. The EBER (EBV encoded small RNA) was not tested. Considering the whole clinical information and history, MTX-associated lymphoma was considered as the final clinical diagnosis. Simple discontinuation of MTX was chosen as a treatment, then 6 months later, all of the multiple liver nodules regressed and diminishment of the ring-like enhancement was confirmed on MRI (Fig. 1A, B). C-reactive protein also showed decrease in value at this time.

RA patients have higher risk of developing lymphoma. MTX is an antimetabolite administered to patients with autoimmune diseases, especially RA. Recently, it was shown that RA patients treated with MTX can develop lymphoproliferative disorders (LPDs) (MTX-LPDs) that share characteristics with the lymphomas. The incidence of MTX-LPDs among patients who are treated with MTX is about 18%.^1^ According to the previous reports, when compared to LPDs patients without MTX treatment (non-MTX-LPDs), the interval between the diagnosis of RA and MTX-LPDs has been reported to be significantly shorter (median 132 months) than that in non-MTX-LPDs (240 months), whereas other clinicopathological features are similar between MTX-LPDs and non-MTX-LPDs.^2^ MTX-LPDs tended to involve the extranodal organs such as gastrointestinal tracts, lung, and skin. On the other hand, exclusive liver involvement is extremely rare to our knowledge.^3,4^

MTX-LPDs of the liver have been reported to show poor enhancement or ring-like enhancement on contrast-enhanced CT, whereas there were few reports describing MRI findings. Our patient’s findings of CT and MRI were consistent with the reported classical features of primary hepatic lymphoma, but they can also be observed in other disease entities including intrahepatic cholangiocarcinoma, metastasis, abscess, and inflammatory pseudotumor.

In conclusion, we reported a rare case of DLBCL solely involving the liver, as determined on PET/CT, in a patient with RA treated with MTX that regressed after simple discontinuation of MTX. Although imaging findings of MTX-LPDs may be relatively non-specific, it is important for radiologists to recognize this specific disease entity, particularly in patients with RA treated with MTX.
